# Impact of a virtual reality-based simulation training for shoulder dystocia on human and technical skills among caregivers: a randomized-controlled trial

**DOI:** 10.1038/s41598-024-57785-6

**Published:** 2024-04-03

**Authors:** Veronica Falcone, Anja Catic, Florian Heinzl, Philipp Steinbauer, Michael Wagner, Fanny Mikula, Tim Dorittke, Bernhard Roessler, Alex Farr

**Affiliations:** 1 Division of Obstetrics and Feto-Maternal Medicine, Department of Obstetrics and Gynecology, Comprehensive Center for Pediatrics, Medical University of Vienna, Waehringer Guertel 18-20, 1090 Vienna, Austria; 2 Division of Neonatology, Pediatric Intensive Care Medicine and Neuropediatrics, Department of Pediatrics, Comprehensive Center for Pediatrics, Medical University of Vienna, Vienna, Austria; 3 Medical Simulation and Emergency Management Research Group, Department of Anaesthesia, Intensive Care Medicine and Pain Medicine, Medical University of Vienna, Vienna, Austria

**Keywords:** Shoulder dystocia, Simulation training, Virtual reality, Health services, Public health

## Abstract

This study analyzed the adherence to the modified Advanced Life Support in Obstetrics (ALSO) algorithm (HELP-RER) for handling shoulder dystocia (SD) using a virtual reality (VR) training modality. Secondary outcomes were improvements in the post-training diagnosis-to-delivery time, human skills factors (HuFSHI), and perceived task-load index (TLX). Prospective, case–control, single-blind, 1:1 randomized crossover study. Participants were shown a 360° VR video of SD management. The control group was briefed theoretically. Both groups underwent HuFSHI and HELP-RER score assessments at baseline and after the manikin-based training. The TLX questionnaire was then administered. After a washout phase of 12 weeks, we performed a crossover, and groups were switched. There were similar outcomes between groups during the first training session. However, after crossover, the control group yielded significantly higher HELP-RER scores [7 vs. 6.5; (*p* = 0.01)], with lower diagnosis-to-delivery-time [85.5 vs. 99 s; (*p* = 0.02)], and TLX scores [57 vs. 68; (*p* = 0.04)]. In the multivariable linear regression analysis, VR training was independently associated with improved HELP-RER scores (*p* = 0.003). The HuFSHI scores were comparable between groups. Our data demonstrated the feasibility of a VR simulation training of SD management for caregivers. Considering the drawbacks of common high-fidelity trainings, VR-based simulations offer new perspectives.

## Introduction

The use of simulation training is well established in various fields of medical education, yielding improved patient safety and improving the outcomes of procedures necessary in emergencies^1^. Recent data suggest that virtual reality (VR) may be used to simulate emergency scenarios in a flexible, safe, and reproducible manner^2^. While the benefits of VR for improving practical skills are to date well known^3,4^, its integration into enhancing non-technical skills (NTS), including coping with stress, teamwork, and conflict resolution, is still limited^3^. On this topic, structured instruments, such as the Human Factors Skills for Healthcare Instrument (HuFSHI)^5^ and the NASA Check-Load Index (TLX)^6^ have been developed and validated to assess self-efficacy of NTS during and after simulation trainings in different settings. The 360-degree technology (360°) is a subset of VR that allows an immersive experience through non-interactive visual and auditory observations^7^. Previous studies have shown higher readiness to perform tasks and higher satisfaction of participants after 360°-VR training than traditional training methods^7–9^. However, there is still limited data on the outcomes of similar training programs in healthcare emergencies. 

Acute intrapartum shoulder dystocia (SD) is one of the most common emergencies in obstetrics. From a practical perspective, SD occurs if the fetal shoulder cannot be delivered with gentle traction to the fetal head, thus requiring further obstetric maneuvers^10^. Because the fetal umbilical cord pH decreases by 0.04 per minute during shoulder dystocia^11^, and the consecutive high risk of neonatal peripartum asphyxia, caregivers must be able to accomplish delivery within a few minutes to avoid permanent sequelae^12,13^. Hence, regular simulation training is required to achieve confidence in hands-on procedures and teamwork and to adapt to exceptional psychological demands during these circumstances^1,14^. No randomized controlled trial has been performed to assess the superiority of one approach to shoulder dystocia management over the other; however, a systematic approach can be integrated into every scenario to reduce the incidence of perinatal complications^10^. At our department, multidisciplinary high-fidelity simulation training involving obstetricians, midwives, anesthesiologists, and neonatologists is performed regularly during a dedicated “simulation week”. However, high-fidelity training is both cost and resource intensive. Moreover, the multidisciplinary character of these mannequin-based training programs prevents the inclusion of many personnel. While high fidelity simulations have long been the standard method for trainings, there is a growing recognition of the advantages offered by the VR technology^4^.

The immersive experience offered by VR technology can increase participants’ confidence while acting in fictious emergencies or to a higher triage accuracy and lesser time needed to solve the given task^2^. This level of immersion enables trainees to practice responding to shoulder dystocia in a highly dynamic and realistic environment. Furthermore, VR trainings offer more flexibility and allows repeated training sessions ad libitum, without being bound by the logistical constraints that are associated with conventional trainings^4^. Hence, the main aim of our trial was the evaluatation of the usefulness of VR on technical and non-technical skills in different healthcare professions during the simulation training of SD management.

## Materials and methods

### Study design, setting, and ethical statements

This prospective, case–control, single-blind, 1:1 randomized crossover study (Fig. 2) was performed at the Obstetric Unit of our University Hospital, between January and August 2022. The study was conducted in accordance with the Declaration of Helsinki and the good clinical practice. The Data Protection Commission of the Medical University of Vienna (Datenschutzkommission der MedUni Wien) approved the clinical trial, and the institutional ethics committee of the Medical University of Vienna issued a waiver of approval. All participants provided written informed consent for enrolment in the study. This trial has been registred in the publicly accessible primary register with the registration number ISRCTN12194978 04/08/2023. This manuscript was structured according to the CONSORT 2010 checklist for randomized controlled trials.

### Participants and recruitment

We contacted all eligible study participants via email and invited them to participate voluntarily in the SD training. Potential study participants included resident and attending physicians, midwives, and medical students in their final year of medical school; these personnel listed were considered eligible for study inclusion and randomization. An important prerequisite for the eligibility for the study was a theoretical understanding of the maneuvers needed to solve a shoulder dystocia scenario. Study participants were informed about the objectives of the study, signed an informed consent form, and completed the validated HuFSHI questionnaire to explore their critical self-reflection before and after training^5^.

### Study groups, randomization, and blinding

**Figure 1:**
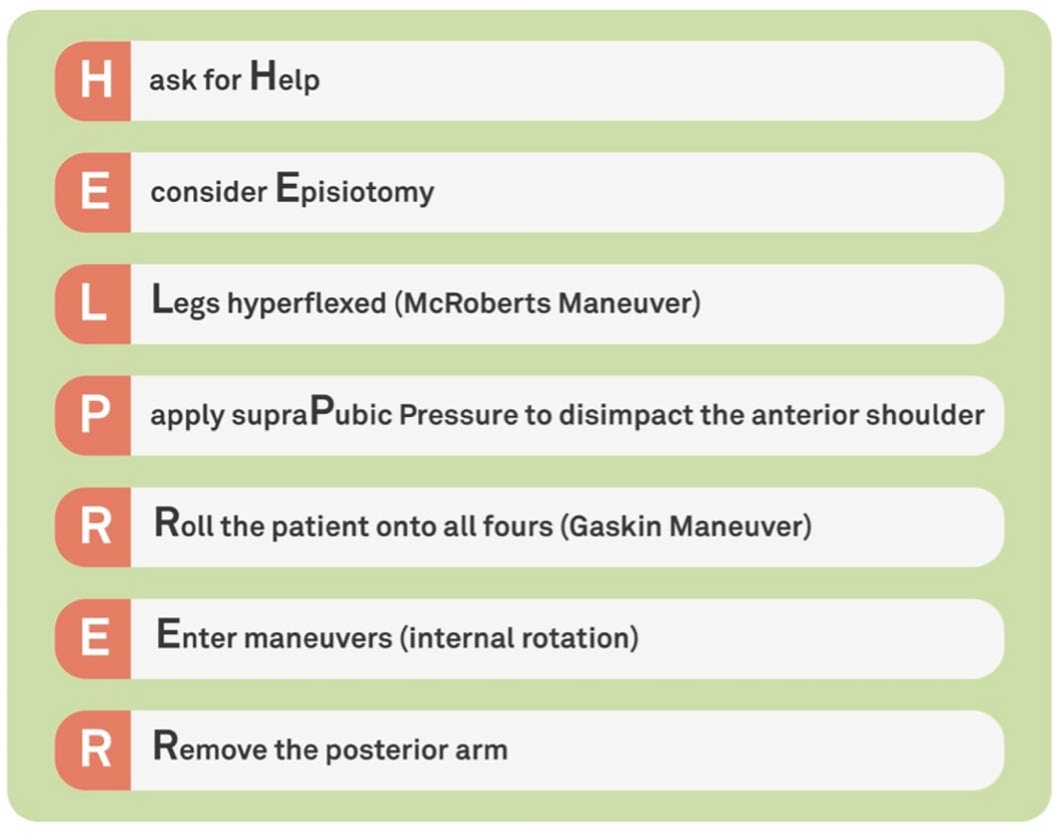
The HELP-RER mnemonic formula, modified from Baxley and Gobbo^32^.

**Figure 2:**
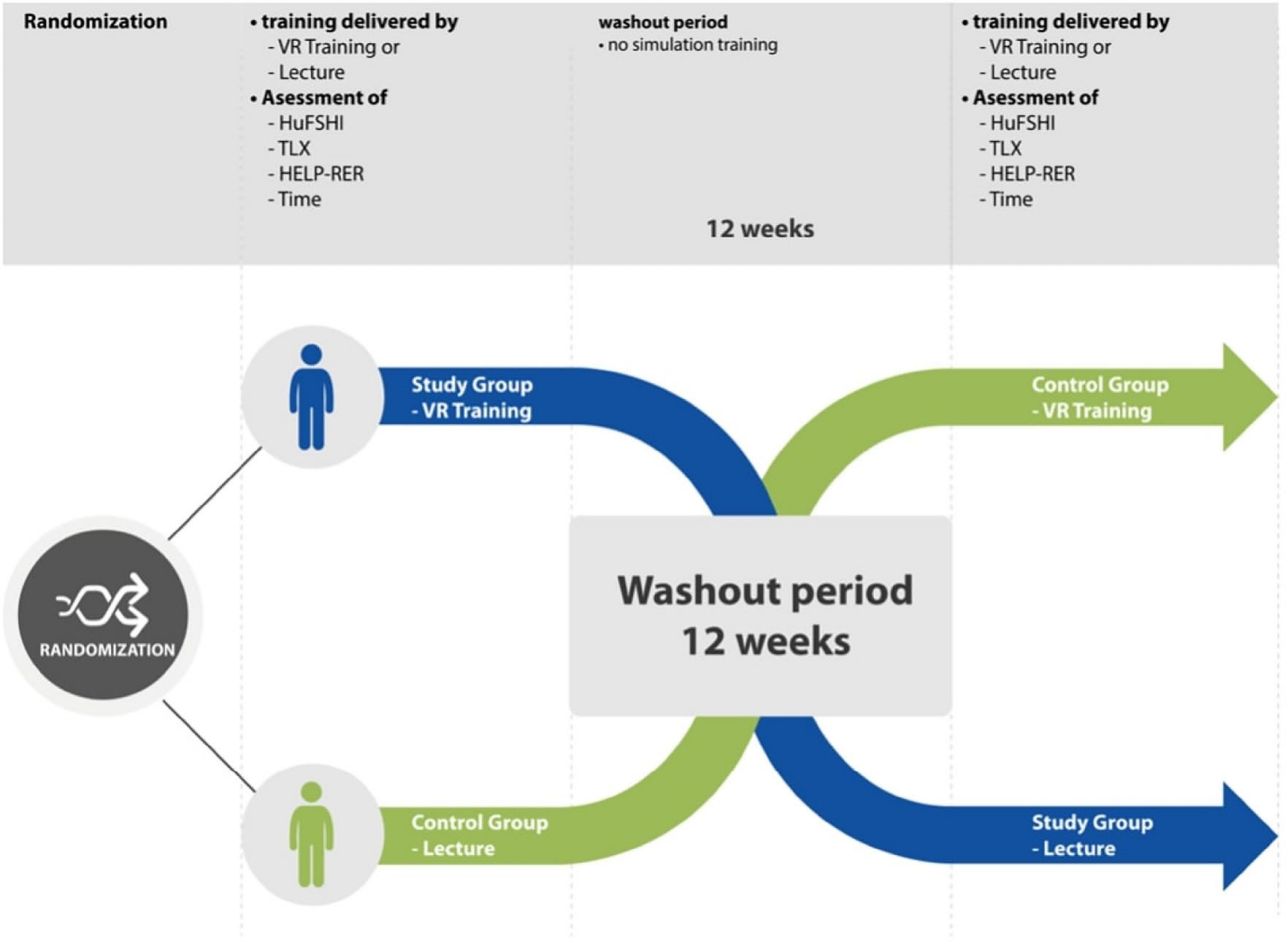
Study design of the prospective case–control, single-blinded, 1:1 randomized cross-over study, involving 61 participants among resident and consultant physicians, midwives and medical students during their final year of medical school.

A blinded sub-investigator at the Obstetric Unit of our University Hospital performed blocked simple randomization with a 1:1 allocation ratio and randomly varying block size. Participants signed an informed consent form and were allocated to either a study group (360° video played on a VR device) or a control group (frontal theoretical lesson via PowerPoint presentation). Sealed envelopes containing information about random allocation to the study (360° video played on a VR device) or control groups (frontal instruction) were supplied according to the randomization sequence. The 360°-VR video scenario for the study participants is described in detail below. For the control group, the second trainer gave a brief lecture on SD management describing 1:1 the shoulder dystocia scenario shown in the 360° video using a PowerPoint presentation (Microsoft, Redmond, Washington, United States) for 2 min and 50 s. Participants in the control group were allowed to ask questions; however, didactic teaching was not allowed. Thus, the trainer did not answer the participants ‘questions directly but tried to create an interactive learning environment to encourage self-directed learning’. The trial envelopes were safely stored at the study site and opened by the trainers during the first training day, allowing participants to be allocated. Each participant completed the HuFSHI, a 14-item questionnaire with a 1–10 Likert scale to assess human factor skills within healthcare providers (Supplemental Material 1)^5^. The HELPERR (H: call for help, E: consider episiotomy, L: legs, P: suprapubic pressure, E: enter maneuvers, R: remove the posterior arm, R: roll onto all fours) mnemonic formula, developed by the Advanced Life Support in Obstetrics (ALSO) providers, can be useful during acute SD management. According to local standards^15^, the Gaskin Maneuver (R) was anticipated and performed in cases where “L” (Mc Roberts maneuver) and “P” (suprapubic pressure) were unsuccessful. Therefore, the HELPERR mnemonic formula was modified for the HELP-RER (H: “call for help”, E: “consider episiotomy”, L: “legs”, P: “suprapubic pressure”, R: “rotate onto all fours”, E: “enter maneuvers”, R: “remove the posterior arm”) checklist, as shown in Fig. 1. The taught content was identical and did not differ between the groups. After training, each participant completed the NASA Task-Load Index (TLX) ^6^, a 6-item questionnaire with a 1 to 21 Likert scale to assess subjective workload during practical training (Supplemental Material 1). The participants and trainers were not blinded to the type of intervention or to the HELP-RER evaluation based on the nature of the study. The principal investigator was blinded to the type of intervention and did not participate in training. The outcome parameters (HuFSHI and TLX, time needed to solve the task, and HELP-RER algorithm score) were documented by the trainers at the end of the first training as HuFSHI(1), Time (1) TLX(1), and HELP-RER(1), and at the end of the second training (HuFSHI(2), time (2) TLX(2), and HELP-RER(2) (Fig. 2).

### The 360°-VR scenario

All training sessions were conducted in the delivery room of the Obstetric Unit of our University Hospital. An immersive 360°-VR scenario was developed to explore all obstetrical maneuvers needed to manage shoulder dystocia and lasted for 2 min and 50 s. The scenario was scripted and approved by a group of experts in the field of obstetrics and produced using a GoPro Max 360 camera (GoPro, San Mateo, California, US) remaining central in the room, thus allowing the 360°fields of view to be accessible by each participant viewing in the Quest 2 VR headset (Meta, Menlo Park, California, US). This headset features a state-of-the-art 845 Snapdragon processor and 4k resolution at a 75-Hz refresh rate (Meta, Menlo Park, California, US). One of the main advantages of this device is its standalone feature, as there is no need to plug it into a computer or use any additional sensors while still offering the option to connect wirelessly to external devices for a live view. The participants in the 360°VR group were allowed to move freely within the delivery room to enhance their immersive VR experience.

**Figure 3:**
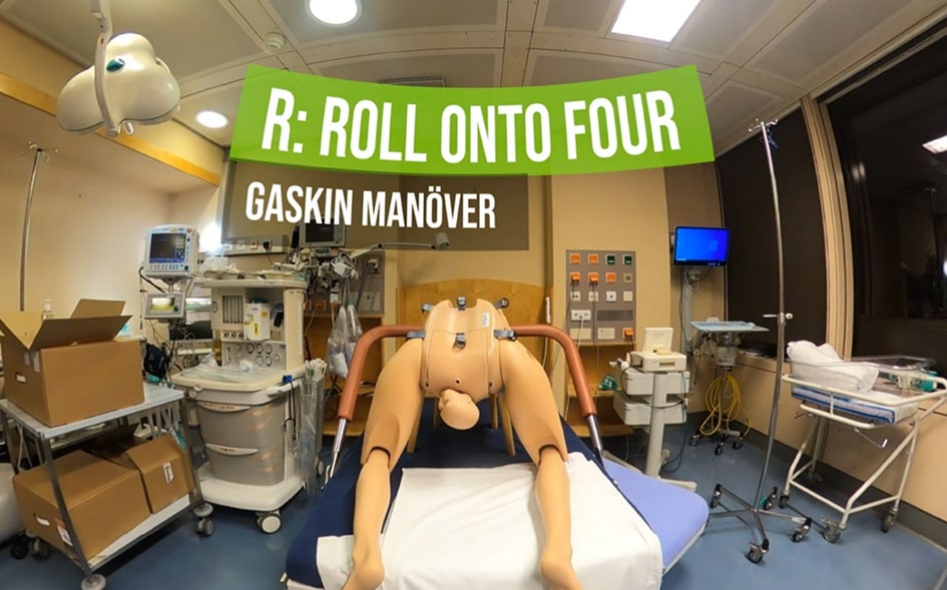
Example of an image shown during the 360° VR video training.

The script of the VR scenario was as follows: A 35-year-old female, Grav 1 Para 0, 160 cm, 85 kg, who had a spontaneous rupture of membranes and regular contractions in gestational week 40 + 2, was presented in the delivery room. She reported an uneventful pregnancy, except for gestational diabetes (treated with lifestyle modifications). According to the last fetal biometry performed at 36 + 0 weeks of gestation, fetal weight was estimated to be at the 70th percentile. Oxytocin infusion was initiated at a cervical dilation of 7 cm. Delivery was assisted by an experienced midwife and an obstetrical/gynecologist (ob/gyn) resident physician. After the delivery of the head, they observe the typical “turtle sign”, thereby defining shoulder dystocia. The resident physician immediately stopped the oxytocin infusion and called for help (H). The need for episiotomy (E) was also evaluated. Subsequently, they performed the McRoberts maneuver three times (L). A second midwife attempted to deliver the baby’s shoulder, but this was unsuccessful. The suprapubic pressure maneuver (P) was also unsuccessful. Subsequently, the obstetric team urged the patient to roll onto all four (R) in order to allow the delivery of the posterior shoulder. During the Gaskin maneuver (Fig. 3), a highly experienced attending physician arrived, and together with the midwife, they rolled the patient on her back to perform the Wood maneuver (E). The baby’s shoulder was delivered, and the baby was eventually transferred to the neonatology ward after immediate cord clamping.

### Assessment and outcomes

Following this first phase, each study participant underwent hands-on training on a high-fidelity PROMPT flex-advanced birth simulator manikin (Limbs and Things Ltd., Bristol, UK). Two different trainers noted the time needed to complete the task as well as the correct execution of the obstetrical maneuvers, as displayed in the modified ALSO algorithm HELP-RER(1). In particular, for each correctly performed letter on this checklist, the participants received one point. Thus, each participant reached a maximum score of 7 on the HELP-RER evaluation. In order to evaluate both short and long time retention through the two different training modalities, as well as to offer all participants both training modalities, each participant underwent alternative training (crossover) after a 12 weeks washout period^16^. HELP-RER scores were reassessed during hands-on training (HELP-RER(2)). The HuFSHI and TLX questionnaires were repeated (Fig. 2). The primary outcome of this study was the evaluation of adherence to the modified ALSO algorithm (HELP-RER) according to the training modality. The secondary outcomes were improvement in the diagnosis-to-delivery time, improvement in HuFSHI, and TLX scores after VR-based versus theory-based training. Further exploratory outcomes included the influence of clinical experience and training reiterations on the final scores.

### Sample size calculation

There was no previous data on training effectiveness upon which to base our sample size calculation. We performed an a priori power analyses (with sample sizes per group ranging from 25 to 30), using the standard assumptions of power = 0.8 and significance levels of 0.05. Hence, a sample size of 32 per intervention was estimated to fit for this assumption.

### Statistical methods

Quantitative variables are presented as the median and interquartile range (IQR), whereas categorical variables are reported as numbers and percentages (%). Associations between numerical outcomes were tested using the Wilcoxon rank-sum test. The chi-square test was used to compare the binary outcomes between participant groups. Statistical significance was set at *p*-values < 0.05 were considered statistically significant. To correct possible confounders, linear regressions with HELP-RER(2) as a dependent variable was performed to investigate the association between higher HELP-RER(2) scores after shoulder dystocia training and the following variables with a potential influence on the outcome: HELP-RER(1), years of clinical expertise, group, and education. For the model without HELP-RER(1), we computed an Akaike information criterion (AIC) of 117.52 vs. an AIC of 116.1 for the full model. Statistical analyses were performed using R software version 4.1.3 (R Development Core Team, Boston, MA, USA)^17^ and the packages pwr^18^ and ggplot2^19^.

### Role of the funding source

This research received no specific grant from any funding agency.

## Results

**Figure 4:**
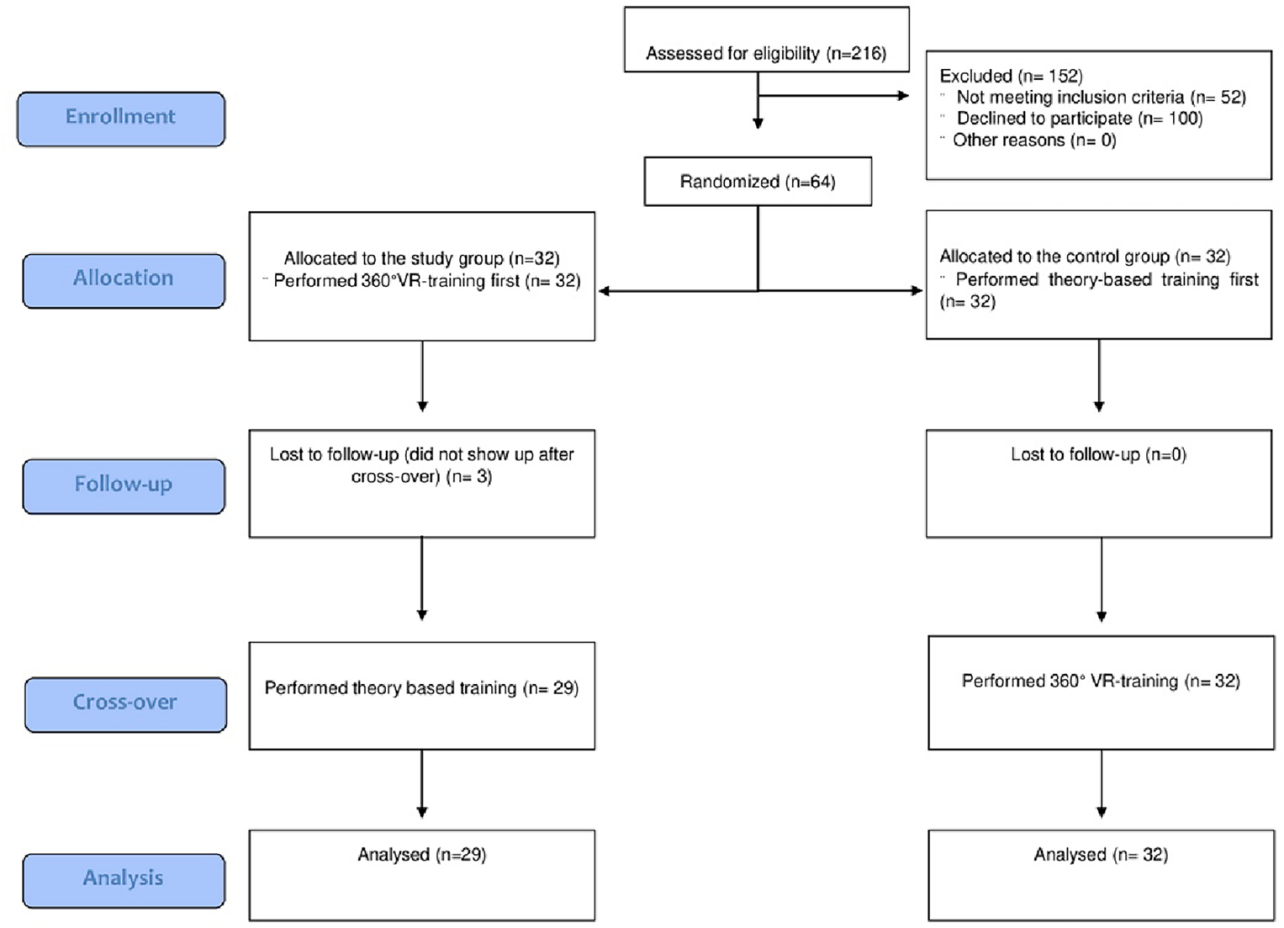
CONSORT flow diagram of the recruited study participants.

We enrolled 64 participants between November and December 2021; 32 (50%) were assigned to the study group and 32 (50%) to the control group. 3 (6.25%) of the 32 participants in the study group did not show up after crossover and were therefore excluded from the final data analysis (Fig. 4). Hence, the data from 61 participants were analyzed and presented in the following section. The baseline characteristics of the study population are summarized in Table 1. Of the 61 participants, 13 (21.3%) were resident physicians, 9 (14.8%) were attending physicians, 18 (29.5%) were midwives, and 18 (29.5%) were medical students in their last year of medical school. Overall, there was no statistically significant difference in the level of training and in the length of time in practice between the study and control groups (Table 1).

**Table 1:** Baseline characteristics of 61 participants being trained for shoulder dystocia by a 360°-VR training (study group) or a theory lecture (control group).

Variable	Study group (n = 29) (%)	Control group (n = 32) (%)	All (%)	*p-*value
Profession				0.63
Resident	5 (17.2)	8 (25)	13 (21.3)	
Consultant	6 (20.7)	3 (9.4)	9 (14.8)	
Midwife	9 (31)	9 (28.1)	18 (29.5)	
Student	9 (31)	9 (28.1)	18 (29.5)	
Gender				1
Female	24 (82.8)	26 (81.3)	50 (82)	
Male	5 (17.2)	6 (18.8)	11 (18)	
Years of clinical expertise				0.85
0–1	14 (48.3)	12 (37.5)	26 (42.6)	
1–5	9 (31)	12 (37.5)	21 (34.4)	
5–10	5 (17.2)	7 (21.9)	12 (19.7)	
> 10	1 (3.4)	1 (3.1)	2 (3.3)	

Data are presented as number (%).

As shown in Table 2, no statistically significant differences for the outcome HELP-RER(1) were identified between the groups (*p* = 0.08).

**Table 2:** Outcomes of 61 participants after training for shoulder dystocia by a 360°-VR training (study group) or a theory lecture (control group).

Variable	Study group (n = 29)	Control group (n = 32)	All	*p-*value
First training				
HuFSHI (1)	86 (79–93)	89 (81.75–95)	88 (80–95)	0.34
TLX (1)	67 (58–83)	67.5(63.25–83)	67 (61–83)	0.44
HELP-RER (1)	6.5 (6–7)	6 (5.5–6.5)	6 (5.5–7)	0.08
Time (1) (s)	103 (87–125)	109.5 (87.5–132.5)	108 (87–132)	0.81
Second training				
HuFSHI (2)	88 (81–94)	90 (82.75–97.25)	88 (81–95)	0.60
TLX (2)	68 (56–79)	57 (43.75–68.75)	65 (50–76)	0.04
HELP-RER (2)	6.5 (6–7)	7 (6–7)	6.5 (6–7)	0.01
Time (2) (s)	99 (75–120)	85·5 (66·5–98·25)	90 (69–108)	0.02

Data are presented as median (IQR).

*VR* virtual reality, *HuFSHI*(1) human factors skills for healthcare instrument during first training, *TLX*(1) NASA Task Load Index after first training, *HELP-RER*(1) modified ALSO algorithm after first training, *HuFSHI*(2) human factors skills for healthcare instrument during second training, *TLX*(2) NASA Task Load Index after second training, *HELP-RER*(2) modified ALSO algorithm after second training.

**Figure 5:**
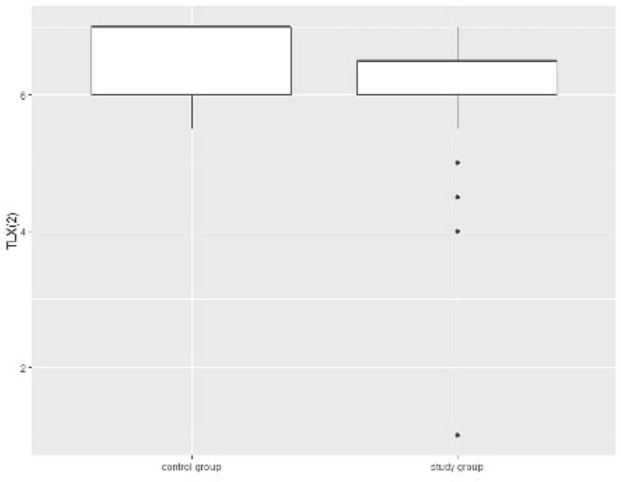
Boxplot for TLX(2) scores among 61 participants being trained by a 360°-VR training (study group) or a theory lecture (control group).

After crossover, the control group underwent VR training, whereas the study group was only theoretically trained. Both groups underwent manikin-based training directly after VR or theoretical lessons. Participants in the control group yielded significantly higher HELP-RER(2) scores [7 (95% CI, 6–7) vs. 6.5 (95% CI, 6–7); *p* = 0.01)], with lower diagnosis-to-delivery-time [85.5 s (95% CI, 66.5–98.25) vs. 99 s (95% CI, 75–120); (*p* = 0.02)] and lower reported subjective workload [57 (95% CI, 43.75–68.75) vs. 68 (95% CI, 56–79); (*p* = 0.04)], as demonstrated on Table 2 and Fig. 5. Notably, the diagnosis-to-delivery time and subjective workload were comparable between the groups after the first training session (Table 2). As shown in Table 3, 29 of the 32 participants (90.6%) in the control group and 14 of the 29 participants (48.3%) in the study group had higher HELP-RER(2) scores after crossover (*p* < 0.001). In total, 15 of 29 participants (51%) in the study group achieved lower HELP-RER(2) scores after the crossover.

**Table 3:** HELP-RER(2) scores of 61 participants after training for shoulder dystocia by a 360°-VR training (study group) or a theory lecture (control group), with respect to HELP-RER(1) scores.

Group	Higher HELP-RER(2) score	Lower HELP-RER(2) score	*p*-value
Control group (n = 32)	29 (90.6%)	3 (9.4%)	< 0.001
Study group (n = 29)	14 (48.3%)	15 (51.7%)	

Data are presented as number (%).

Following univariate analyses, a nested multivariable linear regression model was used to assess the effect of 360°VR on the HELP-RER(2) scores. Interestingly, 360°-VR (*p* = 0.004) and > 10 years of expertise (*p* = 0.03) were independently associated with higher HELP-RER(2) scores (Table 4). Notably, the HELP-RER(1) score was not a statistically significant predictor and did not impact the model itself (Table 4). Additionally, we observed a statistically significant lower meantime needed to solve the task within the control group after crossover [109.5 s (95% CI, 87.5–132.5) vs. 85.50 s (95% CI, 66.50–98.25); (*p* = 0.003)] (Table 5). Notably, the time needed to solve the task was not significantly lower in the study group after crossover [103 s (95% CI, 87–125) vs. 99 s (95% CI, 75–120); (*p* = 0.23)] (Table 5).

**Table 4:** Multivariate linear regression model for achieving higher HELP-RER(2) scores among 61 participants being trained by a 360°-VR training (study group) or a theory lecture (control group).

Variable	Estimates	*p*-value
Male gender	−0.341	0.098
Study group inclusion	−0.506	0.003
1–5 years of clinical expertise	−0.141	0.57
> 10 years of clinical expertise	−0.823	0.044
5–10 years of clinical expertise	−0.174	0.73
Consultant and resident physician	−0.212	0.62
Midwife	−0.202	0.38
Medical student	−0.153	0.55
HELP-RER(1) score	0.071	0.48

HELP-RER(1) modified ALSO algorithm after first training.

**Table 5:** Time needed to solve the task before and after cross-over among 61 participants being trained by a 360°-VR training (study group) or a theory lecture (control group).

Group	Time(1) (s)	Time(2) (s)	*p*-value
Study group (n = 29)	103 (87–125)	99 (75–120)	0.23
Control group (n = 32)	109.5 (87.5–132.5)	85.50 (66.50–98.25)	0.003

Data are presented as median (IQR).

## Discussion

### Main findings

This randomized, controlled crossover trial investigated the effectiveness of VR-based simulation training in an SD scenario for healthcare providers. We found lower diagnosis-to-delivery times and higher HELP-RER scores after crossover among those trained with VR. Furthermore, most participants in the control group achieved higher HELP-RER(2) scores after VR training, independent of sex, educational status, and HELP-RER(1) scores.

Our results reflect the positive effect of a digital learning method based on a concrete experience that confers personal meaning to abstract concepts, as theorized by Kolb^20^. High fidelity trainings are commonly used to train care-givers in emergency situations. Within these settings, technical^21^, and communication skills^22^, as well as trainees’ satisfaction^23^ can be enhanced providing debriefing and feedback after mannequin based training.

However, high-fidelity training is both cost and resource intensive. In contrast, VR training is available on demand for all healthcare providers, as it does not require specific infrastructure, high-fidelity equipment, or trainers.

According to a recent analysis performed by the ACOG simulation working group, there is wide heterogeneity between residency programs and the availability of simulation training^24^. However, there is an overall need for simulation training for rare scenarios or complex obstetric interventions, as highlighted in the literature^24,25^.

Almost two decades ago, Crofts et al. highlighted the benefits of hybrid simulation training for shoulder dystocia in terms of better use of basic maneuvers, higher rates of successful deliveries, and improved communication skills after training^26^. In a subsequent analysis performed 6 and 12 months after the first training session, the participants retrieved the same skills at follow-up^27^. Ten years after regularly performed shoulder dystocia training was introduced, the study group showed a lower rate of shoulder dystocia-related neonatal injury^28^.

The currently available literature underlines that simulation training is crucial for fostering technical and non-technical skills. Hence, the higher HELP-RER scores and shorter time needed to solve the task after VR training highlight the great potential of hybrid simulation training for shoulder dystocia involving VR technology. Furthermore, medical documentation of the diagnosis-to-delivery time and obstetrical maneuvers must be accurate, considering maternal counseling following SD and precautions in subsequent pregnancies. Olson et al. recently reported a strong correlation between shoulder dystocia training and improved clinical documentation, patient counseling, and communication skills among healthcare providers^29^. Communication skills are significantly correlated with improved clinical performance, suggesting that teamwork improves clinical outcomes in emergencies^30^. In our trial, HuFSHI analysis did not reveal statistically significant differences between the groups^5^. However, the VR training was significantly associated with a lower subjective workload, as assessed using the TLX score.

Interestingly, the factor “teamwork” was not stressed within our trial, and this suggests that the participants ‘ability to work as “a team” may not have been adequately assessed or emphasized. In the analysis of NTS, our focus was mainly on adherence to the HELP-RER algorithm. This suggests that other NTS were not specifically targeted in our trial.

Hence, it is important to consider all relevant NTS when designing and conducting studies because they can significantly impact performance and outcomes.

### Clinical and research implications

Our results highlighted the benefits of novel digital teaching methods that can enhance problem-solving skills and reduce anxiety during training. One of the major advantages of 360°-VR training is the opportunity to perform the simulation every time and without any limitations, indicating that this kind of training offers great flexibility. To address the potential skepticism about novel training techniques in medical emergencies, we suggest hybrid shoulder dystocia training, which is also supported by the literature^31^. Therefore, additional research with a larger number of participants is needed to assess the advantages of utilizing hybrid simulation training for shoulder dystocia.

### Strenghts and limitations

To the best of our knowledge, this is the first study involving 360°-VR technology to simulate an SD scenario. One strength of our study is that we implemented a novel technique to perform simulation training in an obstetrical emergency. However, the following limitations need to be mentioned: first, the study design made it impossible to rule out information exchange between the participants of both groups during the one-to-one high-fidelity training. Trainers evaluating the HELP-RER algorithm were not blinded to whether they were evaluating the study or the control group. Moreover, one-to-one training could have influenced trainees’ motivation and outcomes. However, one of the most crucial issues was the absence of a haptic modality during our VR training, which could have significantly influenced the outcomes.

## Conclusions

360°-VR can be implemented within the training modalities for the management of SD in obstetrics. We found a lower diagnosis-to-delivery time, higher fidelity to the modified ALSO algorithm and a lower subjective workload in participants who used VR technology after crossover compared to those who used theory-based training. However, we suggest that the order of training modalities may matter. With immersive 360°-VR, all the distractions that go along with emergencies (real or simulated) can interfere with the cognitive learning process. It is plausible that with 360°-VR used first, learners experienced more cognitive overload and distraction when trying to grasp new concepts, as opposed to the control group, who were afforded simple, step-by-step instruction, followed by a simulation, then the more complex assimilation into 360°-VR and repeated simulation.

### Supplementary Information

Supplementary Information 1.

Supplementary Information 2.

## Data Availability

This research has been reviewed by the Data Protection Commission of our University Hospital, and the institutional review board issued a waiver of approval. After publication, the data and study protocol will be made available to others upon reasonable requests to the corresponding author. A proposal with a detailed descriptions of the study objectives and the statistical analysis plan will be needed for evaluation of the reasonability of requests.
