# Supplementary material for: Impact of a virtual reality-based simulation training for shoulder dystocia on human and technical skills among caregivers: a randomized-controlled trial
Source: Sci Rep. 2024 Apr 3;14:7898. doi: 10.1038/s41598-024-57785-6 (PMC10991516; doi:10.1038/s41598-024-57785-6)
Supplement: Supplementary file 1 — Supplementary Information 1. [file 41598_2024_57785_MOESM1_ESM.pdf]

Questionnaire: Human Factors Skills for Healthcare Instrument (modified from (1))

This is an anonymous survey, and responses are anonymized. No personal data about you will be stored unless explicitly requested (e.g. age, gender).

1. Which gender do you identify with?

a) Male

b) Female

c) Diverse

d) Prefer not to say

2. Which age group do you belong to?

a) 18-24

b) 25-29

c) 30-38

d) 39-49

e) 50-59

f) > 59

3. How constructively do you deal with negative emotions towards your colleagues? (0 not good at all, 10 extremely well)

0 • 1 • 2 • 3 • 4 • 5 • 6 • 7 • 8 • 9 • 10 •

4. You can effectively communicate with colleagues who have different opinions than yours (0 = not at all, 10 = extremely effective)

0 • 1 • 2 • 3 • 4 • 5 • 6 • 7 • 8 • 9 • 10 •

5. You can prioritize well when multiple situations occur simultaneously (0= not at all, 10= very well)

0 • 1 • 2 • 3 • 4 • 5 • 6 • 7 • 8 • 9 • 10 •

6. You can ask for help from individuals in other professional groups (0 = not at all, 10 = very easily)

0 • 1 • 2 • 3 • 4 • 5 • 6 • 7 • 8 • 9 • 10 •

7. You can effectively communicate your opinion about the entire situation to the entire team (0 = not at all, 10 = very well)

0 • 1 • 2 • 3 • 4 • 5 • 6 • 7 • 8 • 9 • 10 •

8. You are willing to involve your colleagues in your decision-making process (0 = never, 10 = always):

0 • 1 • 2 • 3 • 4 • 5 • 6 • 7 • 8 • 9 • 10 •

9. You can deal well with your uncertainties during the decision-making process (0 = not at all, 10 = very well):

0 • 1 • 2 • 3 • 4 • 5 • 6 • 7 • 8 • 9 • 10 •

10. Even in a hectic situation, you can ask your colleagues for the important information you need (0 = never, 10 = always)

0 • 1 • 2 • 3 • 4 • 5 • 6 • 7 • 8 • 9 • 10 •

11. You recognize the moment when you need to take leadership (0 = never, 10 = always)

0 • 1 • 2 • 3 • 4 • 5 • 6 • 7 • 8 • 9 • 10 •

12. You can see the "big picture" of a complex clinical situation (0 = always, 10 = never)

0 • 1 • 2 • 3 • 4 • 5 • 6 • 7 • 8 • 9 • 10 •

13. You can anticipate the next steps in a complex clinical situation (0 = never, 10 = always)

0 • 1 • 2 • 3 • 4 • 5 • 6 • 7 • 8 • 9 • 10 •

14. You work effectively even in a team where you don't know the employees well (0 = not at all, 10 = very well)

0 • 1 • 2 • 3 • 4 • 5 • 6 • 7 • 8 • 9 • 10 •

1. Reedy GB, Lavelle M, Simpson T, Anderson JE. Development of the Human Factors Skills for Healthcare Instrument: a valid and reliable tool for assessing interprofessional learning across healthcare practice settings. BMJ Simul Technol Enhanc Learn. 2017 Oct;3(4):135–41.

Figure 8.6

NASA Task Load Index

Hart and Staveland's NASA Task Load Index (TLX) method assesses work load on five 7-point scales. Increments of high, medium and low estimates for each point result in 21 gradations on the scales.

Name	Task	Date

Mental Demand — How mentally demanding was the task?

Very Low — Very High

Physical Demand — How physically demanding was the task?

Very Low — Very High

Temporal Demand — How hurried or rushed was the pace of the task?

Very Low — Very High

Performance — How successful were you in accomplishing what you were asked to do?

Perfect — Failure

Effort — How hard did you have to work to accomplish your level of performance?

Very Low — Very High

Frustration — How insecure, discouraged, irritated, stressed, and annoyed wereyou?

Very Low — Very High
