# Supplementary material for: Impact of a virtual reality-based simulation training for shoulder dystocia on human and technical skills among caregivers: a randomized-controlled trial
Source: Sci Rep. 2024 Apr 3;14:7898. doi: 10.1038/s41598-024-57785-6 (PMC10991516; doi:10.1038/s41598-024-57785-6)
Supplement: Supplementary file 2 — Supplementary Information 2. [file 41598_2024_57785_MOESM2_ESM.pdf]

Fragebogen: Human factors Skills for Healthcare Instrument (modified from [1])

Dies ist eine anonyme Umfrage, die Umfrageantworten sind anonymisiert. Es werden keine persönlichen Daten über Sie gespeichert, es sei denn, es wird ausdrücklich danach gefragt (z.B. Alter, Geschlecht)

1) Welchem Geschlecht fühlen Sie sich gehörig?

   a) Männlich
   b) Weiblich
   c) Divers
   d) Keine Angabe

2) Welcher Altersgruppe gehören Sie?

   a) 18-24
   b) 25-29
   c) 30-38
   d) 39-49
   e) 50-59
   f) > 59

3) Wie konstruktiv gehen Sie mit den negativen Emotionen Ihren Kolleginnen um? (0 gar nicht gut, 10 extrem gut)

   0 • 1 • 2 • 3 • 4 • 5 • 6 • 7 • 8 • 9 • 10 •

4) Sie können gut und effektiv mit Kolleginnen kommunizieren, die nicht Ihrer gleichen Meinung sind (0 = gar nicht, 10 = extrem effektiv)

   0 • 1 • 2 • 3 • 4 • 5 • 6 • 7 • 8 • 9 • 10 •

5) Sie können gut Priorität setzen, wenn mehrere Situationen gleichzeitig geschehen (0= gar nicht, 10= sehr gut)

   0 • 1 • 2 • 3 • 4 • 5 • 6 • 7 • 8 • 9 • 10 •

6) Sie können Personen aus anderen Berufsgruppen nach ihrer Hilfe bitten (0 = gar nicht, 10 = ganz unproblematisch)

   0 • 1 • 2 • 3 • 4 • 5 • 6 • 7 • 8 • 9 • 10 •

7) Sie können gut Ihre Meinung über die gesamte Situation dem gesamten Team übermitteln (0 = gar nicht, 10 = ich kann es sehr gut)

   0 • 1 • 2 • 3 • 4 • 5 • 6 • 7 • 8 • 9 • 10 •

8) Sie sind bereit Ihre Kolleginnen, in Ihren Entscheidungsprozess zu involvieren (0 = nie, 10 = immer):

0 • 1 • 2 • 3 • 4 • 5 • 6 • 7 • 8 • 9 • 10 •

9) Sie können gut mit Ihren Unsicherheiten während des Entscheidungsprozesses umgehen (0 = gar nicht, 10 = sehr gut):

0 • 1 • 2 • 3 • 4 • 5 • 6 • 7 • 8 • 9 • 10 •

10) Auch in einer hektischen Situation können Sie Ihre Kolleginnen nach den wichtigen Informationen fragen, die Sie brauchen (0 = nie, 10 = immer)

0 • 1 • 2 • 3 • 4 • 5 • 6 • 7 • 8 • 9 • 10 •

11) Sie erkennen den Moment, an dem Sie die Leadership übernehmen müssen (0 = nie, 10 = immer)

0 • 1 • 2 • 3 • 4 • 5 • 6 • 7 • 8 • 9 • 10 •

12) Sie können das „gesamte Bild" einer komplexen klinischen Situation sehen (0 = immer, 10 = nie)

0 • 1 • 2 • 3 • 4 • 5 • 6 • 7 • 8 • 9 • 10 •

13) Sie können die nächsten Schritte bei einer komplexen klinischen Situation vorhersehen (0 = nie, 10 = immer)

0 • 1 • 2 • 3 • 4 • 5 • 6 • 7 • 8 • 9 • 10 •

14) Sie arbeiten effektiv auch in einem Team, in dem Sie die Mitarbeiter nicht gut kennen (0 = gar nicht, 10 = sehr gut)

0 • 1 • 2 • 3 • 4 • 5 • 6 • 7 • 8 • 9 • 10 •

Quellenangabe:

1 Reedy GB, Lavelle M, Simpson T, *et al.* Development of the Human Factors Skills for Healthcare Instrument: a valid and reliable tool for assessing interprofessional learning across healthcare practice settings. *BMJ Simul Technol Enhanc Learn* 2017;**3**:135–41. doi:10.1136/bmjstel-2016-000159

Figure 8.6

NASA Task Load Index

Hart and Staveland's NASA Task Load Index (TLX) method assesses work load on five 7-point scales. Increments of high, medium and low estimates for each point result in 21 gradations on the scales.

Name	Task	Date

Mental Demand — How mentally demanding was the task?

Very Low — Very High

Physical Demand — How physically demanding was the task?

Very Low — Very High

Temporal Demand — How hurried or rushed was the pace of the task?

Very Low — Very High

Performance — How successful were you in accomplishing what you were asked to do?

Perfect — Failure

Effort — How hard did you have to work to accomplish your level of performance?

Very Low — Very High

Frustration — How insecure, discouraged, irritated, stressed, and annoyed wereyou?

Very Low — Very High
